# Detection of malignant lesions in cytologically indeterminate thyroid nodules using a dual-layer spectral detector CT-clinical nomogram

**DOI:** 10.3389/fonc.2024.1357419

**Published:** 2024-05-28

**Authors:** Xiaofang Ren, Jiayan Zhang, Zuhua Song, Qian Li, Dan Zhang, Xiaojiao Li, Jiayi Yu, Zongwen Li, Youjia Wen, Dan Zeng, Xiaodi Zhang, Zhuoyue Tang

**Affiliations:** ^1^ Department of Radiology, Affiliated Hospital of Southwest Medical University, Luzhou, China; ^2^ Department of Radiology, Chongqing General Hospital, Chongqing, China; ^3^ Department of Clinical and Technical Support, Philips Healthcare, Chengdu, China

**Keywords:** thyroid nodule, cytology, multidetector computed tomography, nomograms, diagnosis

## Abstract

**Purpose:**

To evaluate the capability of dual-layer detector spectral CT (DLCT) quantitative parameters in conjunction with clinical variables to detect malignant lesions in cytologically indeterminate thyroid nodules (TNs).

**Materials and methods:**

Data from 107 patients with cytologically indeterminate TNs who underwent DLCT scans were retrospectively reviewed and randomly divided into training and validation sets (7:3 ratio). DLCT quantitative parameters (iodine concentration (IC), NIC_P_ (IC nodule/IC thyroid parenchyma), NIC_A_ (IC nodule/IC ipsilateral carotid artery), attenuation on the slope of spectral HU curve and effective atomic number), along with clinical variables, were compared between benign and malignant cohorts through univariate analysis. Multivariable logistic regression analysis was employed to identify independent predictors which were used to construct the clinical model, DLCT model, and combined model. A nomogram was formulated based on optimal performing model, and its performance was assessed using receiver operating characteristic curve, calibration curve, and decision curve analysis. The nomogram was subsequently tested in the validation set.

**Results:**

Independent predictors associated with malignant TNs with indeterminate cytology included NIC_P_ in the arterial phase, Hashimoto’s Thyroiditis (HT), and BRAF V600E (all *p* < 0.05). The DLCT-clinical nomogram, incorporating the aforementioned variables, exhibited superior performance than the clinical model or DLCT model in both training set (AUC: 0.875 vs 0.792 vs 0.824) and validation set (AUC: 0.874 vs 0.792 vs 0.779). The DLCT-clinical nomogram demonstrated satisfactory calibration and clinical utility in both training set and validation set.

**Conclusion:**

The DLCT-clinical nomogram emerges as an effective tool to detect malignant lesions in cytologically indeterminate TNs.

## Introduction

Thyroid nodules (TNs) are highly prevalent, being detected in up to 65% of the general population (1). The nature of TN is the primary concern for both patients and clinicians, as it defines subsequent clinical management. Ultrasound-guided fine-needle aspiration biopsy (US-FNAB) has been regarded as the preoperative standard method for assessing the state of TN (2). However, approximately 10–25% of patients who underwent US-FNAB had cytologically indeterminate TNs, including Bethesda categories III, IV, and V with risks of malignancy of 10–30%, 25–40%, and 50–75%, respectively (2–4). This subset of patients would be recommended for diagnostic lobectomy due to the inability to rule out cancer, with an inevitable consequence of unnecessary and potentially harmful surgery in benign nodules or completion thyroidectomy following the initial diagnostic lobectomy in malignant nodules (2). Studies have shown that approximately 60% of these patients underwent unnecessary surgery (5). Therefore, a reliable preoperative method that could better detect malignant lesions in cytologically indeterminate TN is sorely needed for appropriate clinical management to reduce the burden on patients and health systems.

Some potential imaging tools have been investigated in the differential diagnosis of cytologically indeterminate TN, such as US and positron emission tomography (PET)/CT (6–8). US is the primary imaging method for risk stratification of TN with the advantage of being accessible, inexpensive, and nonradiative (9). However, US has some limitations in assessing the nature of TN due to similarities in ultrasound features between benign and malignant lesions (10), diminished objectivity for operator dependence (11), and various diagnostic standards between different US risk stratification systems (12). (18F)PET-CT showed a high predictive value for malignancy (13), but it is expensive and not widely available. Thus, an objective, accessible, and reliable tool for the proper management of cytologically indeterminate TN is still needed.

Dual-layer detector spectral CT (DLCT) is an advanced functional imaging technique that could reflect valuable hemodynamic and tissue characterization for lesion detection by offering multiparametric data not obtained by conventional CT imaging (14, 15). This method has the advantage of increasing the sensitivity and qualitative accuracy of lesion detection and minimizing metallic artifacts (16). Frequently used DLCT-derived quantitative parameters, including iodine concentration (IC), the slope of the spectral HU curve (λ_HU_), and effective atomic number (Z_eff_), have demonstrated diagnostic potential value in oncologic applications (17, 18). Additionally, previous studies have supported the use of DLCT for differentiating between benign TN and thyroid cancer and evaluating lymph node metastases in thyroid cancer (19, 20). However, the value of quantitative parameters from DLCT in detecting malignant lesions in cytologically indeterminate TN remains elusive.

In this study, we hypothesize that DLCT-derived parameters may be a reliable method to detect malignant TNs with indeterminate cytology. To test our hypothesis, we sought to develop a nomogram combining DLCT parameters and clinical variables to detect malignant TNs in cytologically indeterminate TNs to reduce the need for diagnostic surgery.

## Materials and methods

### Patients selection

This retrospective study received approval from the institutional review board of Chongqing General Hospital, and the written informed consent requirement was waived.

**Figure 1:**
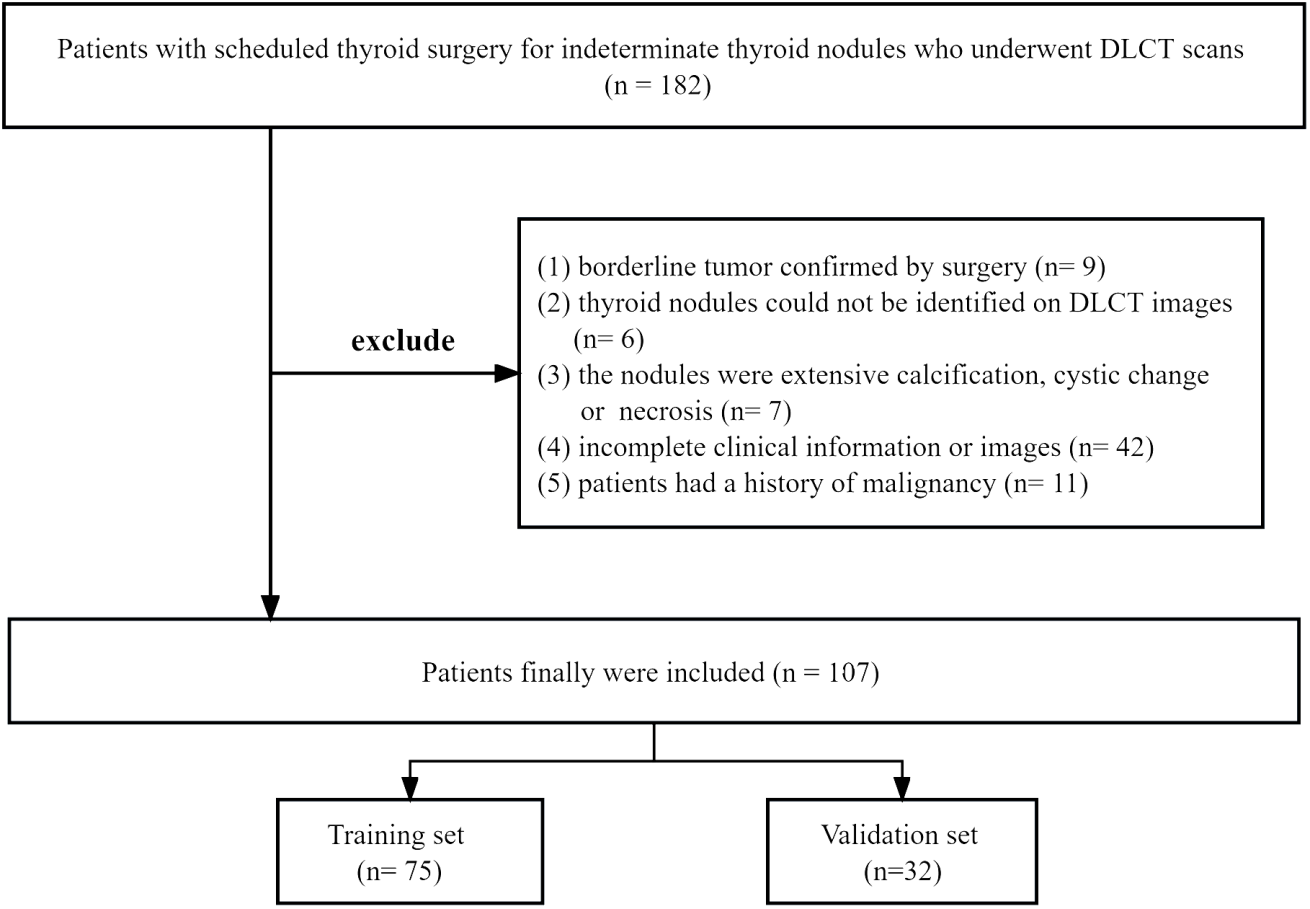
The flowchart of patient selection. DLCT, dual-layer spectral detector computed tomography.

Between August 2021 and January 2023, we reviewed the records of 182 patients who underwent DLCT scans and thyroid surgery for indeterminate thyroid nodules. Exclusion criteria were: (1) borderline tumor confirmed by surgery; (2) thyroid nodules could not be identified on DLCT images; (3) the nodules were extensive calcification, cystic change or necrosis; (4) incomplete clinical information or images; (5) patients had a history of malignancy. Out of the 182 patients initially considered, 75 were excluded. Ultimately, 107 TNs with indeterminate cytology from 107 patients were included and randomly divided into training set and validation set at a ratio of 7:3. All of them were finally pathologically confirmed after surgery. The training cohort was used for model building, while the validation cohort was used for internal validation of the model. The flowchart of patient selection was shown in **Figure 1**.

### Image acquisition

All patients in our study underwent preoperative contrast-enhanced neck scans through 64-slice DLCT devices (IQon Spectral CT, Philips Healthcare). The DLCT scanning protocol was as follows: tube voltage, 120 kVp; tube current, modulated with automated exposure control (DoseRight system, Philips Healthcare); detector collimation, 64 × 0.625 mm; field of view, 350 mm; matrix, 512 × 512; layer thickness, 3 mm; reconstruction thickness, 0.67 mm. Patients were intravenously injected with nonionic contrast media (Iopamidol 350 mg/ml, Bracco) at a dose of 1.5 ml/kg and an injection rate of 3.5 ml/s, followed by 30 ml of saline flashing at the same rate. The arterial phase (AP) scan was performed with a delay of 6 seconds and a venous phase (VP) scan was performed with a delay of 50 seconds after the CT value of the descending aorta lumen at the tracheal bifurcation level reached 150 Hounsfield units, respectively.

### Indeterminate TNs identification

The location and size of indeterminate TNs were used as matching information to correlate nodules seen on US**-**guided FNAB with CT images. The specific matching method was as follows: (1) The location (left lobe, right lobe, isthmus and superior, middle, inferior) of indeterminate TNs were acquired according to the cytologic pathology report; (2) The indeterminate TNs on CT images were identified by a radiologist who referred to the cytologic pathology report; (3) If several nodules lay close to one another, they were discriminated by size acquired from the US-FNAB report.

### DLCT quantitative analysis

DLCT images were transferred from the picture archiving and communication system to a spectral CT postprocessing workstation (IntelliSpace Portal Version 10.1, Philips Healthcare) and were reconstructed into iodine image, monochromatic image, and effective atomic number image for subsequent quantitative analysis.

The approach for quantitative analysis is displayed as follows: First, radiologists enlarged images for accurately measuring lesions, ensuring a clear image. Secondly, circular regions of interest (ROIs) were manually drawn as large as possible on the solid area of the TN on axial iodine images avoiding calcification, cystic or necrotic regions, and additional ROIs of thyroid tissues and that of the ipsilateral carotid artery on the cross-sectional map were selected for normalization. Meanwhile, ROIs were automatically propagated to monochromatic images and effective atomic numbers. All quantitative parameters were measured twice to calculate the average. The measurements were taken by two independent observers trained in head and neck imaging who were only aware of the location of the lesion.

Quantitative parameter-derived DLCT included IC of TN, thyroid parenchyma, and ipsilateral carotid artery, CT values on 40 keV and 100 keV monoenergetic images, and Z_eff_. All quantitative parameters were measured in the AP and VP. To minimize variations caused by the patient’s circulation or hormone status, the IC values of the ipsilateral adjacent artery and thyroid parenchyma were measured to derive the following ratios respectively: NIC_A_ = IC nodule/IC ipsilateral carotid artery, NIC_P_ = IC nodule/IC thyroid parenchyma. CT values on the monoenergetic images were measured to derive the following ratio: λ_HU_ = (HU 40 keV – HU100 keV)/ (100 – 40).

### Clinical data

Clinical variables were included in this study, such as age, sex, BRAF V600E, HT, and body mass index (BMI). BRAF V600E mutation was assessed by FNAB or the medical record. HT was diagnosed according to US and anti-thyroid antibodies (TPOAb > 30 IU/L or TgAb > 95 IU/L) (21). BMI was calculated by the following ratio: BMI = weight (kg)/height (m)^2^.

### Development and validation of nomogram

Statistically significant clinical variables and DLCT quantitative parameters between the benign nodule cohort and malignant nodule cohort were first identified using univariate analysis. Next, the significant variables from the univariate analysis were entered into multivariable logistic stepwise regression analyses to identify independent predictors for malignant TN with indeterminate cytology and to estimate their respective regression coefficients. Afterward, a DLCT-clinical model was developed, incorporating all selected independent predictors weighted by their respective regression coefficients. Similarly, a clinical model was constructed using independent clinical variables and a DLCT model was constructed using independent DLCT quantitative parameters.

The diagnostic ability of the three aforementioned models was evaluated through receiver operating characteristic curve and was tested in the validation set. Sensitivity and specificity were calculated. Decision curve analysis was adopted to assess the clinical utility of the three different models in both the training set and validation set. Subsequently, a nomogram was constructed from the model with optimal diagnostic performance. The calibration curve was applied to evaluate the fit goodness of the nomogram.

### Statistical analysis

All statistical analyses were performed using R software (http://www.R-project.org) and SPSS software (version 25.0, SPSS, IBM). For all DLCT parameters, interobserver reproducibility was assessed with intraclass correlation coefficients (ICC). The Shapiro-Wilk test was used to determine the distribution of continuous data. Normally distributed data were expressed as Mean ± Standard Deviation and assessed by a two-sample t-test, while non-normally distributed data were presented as Median (interquartile range, IQR) and analyzed by the Mann-Whitney U test. Categorical variables were presented as raw numbers, and analyzed by a chi-squared test. Multivariable logistic stepwise regression analyses were performed in the training set to identify independent predictors applied to prediction models and to estimate their odds ratio (OR) and a 95% confidence interval (CI). The stopping rule for forward stepwise selection was the likelihood ratio test with Akaike’s information criterion. A two-sided *p-*value < 0.05 indicated statistical significance.

## Results

### Patient characteristics

A total of 107 histologically confirmed TNs from 107 patients, comprising 44 benign and 63 malignant nodules, qualified for the final analysis of the study. According to the pathological results, the benign nodules included 32 nodular goiters, 3 follicular adenomas, 3 adenomatous nodular goiters, and 6 inflammatory nodules. The malignant nodules included 63 papillary carcinomas.

### Univariate analysis and multivariable analysis

Characteristics of patients in the training and validation sets are detailed in **Table 1**. No significant differences in demographic and clinical characteristics were observed between the two sets (all *p* > 0.05).

**Table 1:** Characteristics of patients in the training and validation sets.

Characteristics	Training set (n = 75)	Validation set (n = 32)	*P* value
Age*	44.44 ± 11.46	46.63 ± 11.89	0.374
Sex			0.256
Male	8	6	
Female	67	26	
BMI (kg/m^2^)^#^	22.27 (20.96, 23.88)	22.31 (20.52, 24.72)	0.951
BRAF V600E			0.093
mutant	29	18	
wild	46	14	
HT			0.533
yes	33	12	
no	42	20	

Unless otherwise indicated, data are the number of patients. ^*^ data are mean ± standard deviation. **^#^** data are median with the interquartile range in parentheses. BMI, body mass index; HT, Hashimoto′s thyroiditis.

Interclass correlation analysis for DLCT parameters showed good concordance between inter- and intraobserver agreements (ICC > 0.75). In the training set, DLCT parameters and clinical variables between the benign and malignant nodule cohorts are summarized in **Table 2**. Univariate analysis revealed significant differences in DLCT parameters (including IC_nodule_, NIC_P_, NIC_A_, λ_HU,_ and Z_eff_ in the AP and VP), BRAF V600E and HT between benign and malignant nodule cohorts (all *p* < 0.05). However, no significant differences were found in age (*p* = 0.171) between the benign cohort (mean age ± standard, 46.48 ± 11.71 years) and malignant cohort (mean age ± standard deviation, 42.83 ± 11.13 years) or in BMI (*p* = 0.806) between the benign cohort (median with the interquartile range, 21.97 with 21.03 - 24.05 kg/m^2^) and malignant cohort (median with the interquartile range, 22.32 with 20.83 - 23.90 kg/m^2^). Multivariable logistic stepwise regression analysis in the training set identified AP-NIC_p_ (OR = 0.004; 95% CI = 0.000 - 0.106; *p* < 0.001), HT (OR = 3.802; 95% CI = 1.037 - 13.933; *p* = 0.044), and BRAF V600E (OR = 6.436; 95% CI = 1.618 - 25.605; *p* = 0.008) as variables independently associated with malignancy (**Table 3**).

**Table 2:** Univariate analysis in the benign and malignant nodule cohort .

Variables	Benign nodule cohort (n = 33)	Malignant nodule cohort (n = 42)	F value/Z value/X^2^value	*p* value
Age^*^	46.48 ± 11.71	42.83 ± 11.13	0.012	0.171
BMI (kg/m^2^)	21.97 (21.03, 24.05)	22.32 (20.83, 23.90)	-0.246	0.806
BRAF V600E^#^			13.740	<0.001
mutant	5	24		
wild	28	18		
HT^#^			4.487	0.034
yes	10	23		
no	23	19		
AP				
IC_nodule_ (mg/ml)	3.84 (3.39, 4.35)	2.33 (1.74, 2.92)	-4.910	<0.001
NIC_P_	0.84 (0.67, 0.93)	0.53 (0.40, 0.71)	-4.792	<0.001
NIC_A_	0.40 (0.32, 0.46)	0.24 (0.18, 0.32)	-5.113	<0.001
λ_HU_	4.66 (3.78, 5.38)	2.88 (2.15, 3.64)	-4.531	<0.001
Z_eff_^*^	9.11 ± 0.41	8.59 ± 0.46	1.029	<0.001
VP				
IC_nodule_^*^ (mg/ml)	3.15 ± 0.88	2.70 ± 0.73	1.005	0.019
NIC_P_	0.86 (0.74, 0.94)	0.74 (0.65, 0.87)	-2.636	0.008
NIC_A_^*^	0.81 ± 0.20	0.67 ± 0.22	0.106	0.008
λ_HU_^*^	3.88 ± 1.19	3.32 ± 0.90	1.399	0.026
Z_eff_^*^	8.85 ± 0.36	8.67 ± 0.32	0.449	0.021

Unless otherwise indicated, data are median with the interquartile range in parentheses. ^*^ data are mean ± standard deviation. **^#^** data are the number of patients.

BMI, body mass index; HT, Hashimoto′s thyroiditis; AP, arterial phase; VP, venous phase; IC_nodule_, iodine concentration of thyroid nodule; NIC_P,_ IC_nodule/_IC_thyroid parenchyma_; NIC_A,_ IC_nodule_/IC_carotid artery_; λ_HU_, slope of the spectral Hounsfield unit curve; Z_eff,_ effective atomic number.

**Table 3:** Stepwise multivariate logistic regression analysis of predictors associated with malignant thyroid nodule with indeterminate cytology in the training set.

Variables	β value	OR	95%CI	*p* value
AP-NIC_P_	-5.540	0.004	0.001-0.106	0.001
HT	1.335	3.802	1.037-13.933	0.044
BRAF V600E	1.862	6.436	1.618-25.605	0.008

β, regression coefficient; OR, odds ratios; CI, confidence interval; AP, arterial phase; NIC_P_, ICnodule/ICthyroid parenchyma; HT, Hashimoto′s thyroiditis.

### Predictive model analysis

**Figure 2:**
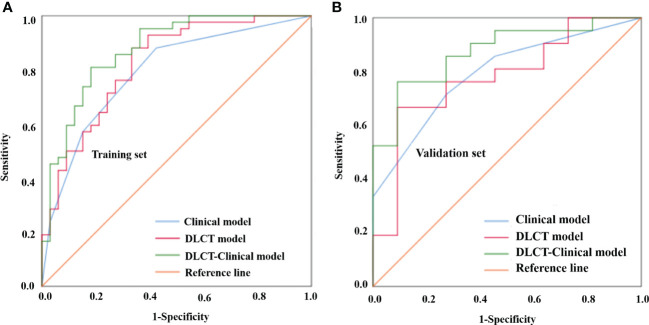
Comparison of the different models for distinguishing benign from malignant thyroid nodules with indeterminate cytology in the training set **(A)** and validation set **(B)**. DLCT, dual-layer spectral detector computed tomography.

Based on the selected predictors from multivariable logistic stepwise regression analysis, three predictive models were established: one clinical model (HT + BRAF V600E), one DLCT model (AP-NIC_p_), and one DLCT-clinical combined model (HT + BRAF V600E + AP-NIC_p_). The classification performance of the three models was shown in **Table 4** and **Figure 2**. The AUC values were 0.792 (95% CI, 0.689 - 0.896) in the clinical model, 0.824 (95% CI, 0.729 - 0.919) in the DLCT model, and 0.875 (95% CI, 0.795 - 0.955) in the combined model for the training set. In the validation set, the AUC values were 0.792 (95% CI, 0.634 - 0.950) in the clinical model, 0.779 (95% CI, 0.608 - 0.950) in the DLCT model, and 0.874 (95% CI, 0.752 - 0.997) in the combined model.

**Table 4:** Results of three models’ predictive ability for detecting malignant thyroid nodules with indeterminate cytology.

Groups	Models	AUC	95CI%	Sensitivity	Specificity
Training	Clinical model	0.792	0.689-0.896	0.881	0.576
	DLCT model	0.824	0.729-0.919	0.786	0.667
	DLCT-clinical model	0.875	0.795-0.955	0.833	0.727
Validation	Clinical model	0.792	0.634-0.950	0.857	0.545
	DLCT model	0.779	0.608-0.950	0.762	0.636
	DLCT-clinical model	0.874	0.752-0.997	0.857	0.727

AUC, area under the curve; CI, confidence interval; DLCT, dual-layer spectral detector computed tomography.

### Development and evaluation of the predictive nomogram

**Figure 3:**
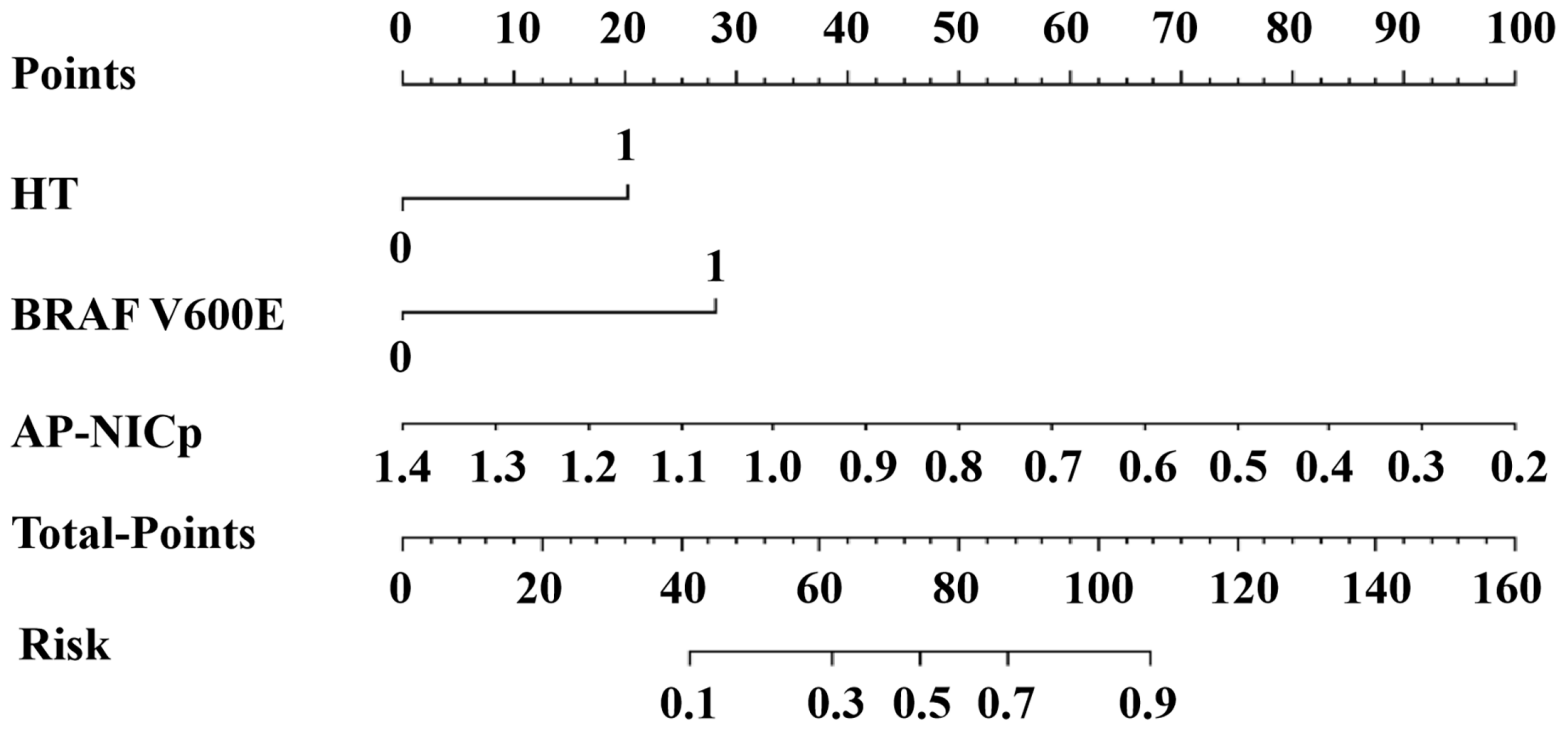
The DLCT-clinical nomogram for assessing malignant nodule risk. AP, arterial phase; NIC_P,_ ICnodule/ICthyroid parenchyma; HT, Hashimoto’s thyroiditis.

**Figure 4:**
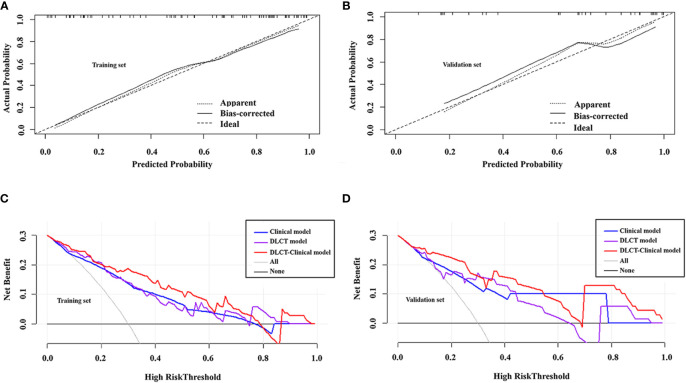
Calibration curves of the DLCT-clinical nomogram in the training set **(A)** and validation set **(B)**. Decision curve analysis of the three models in the training set **(C)** and validation set **(D)**.

**Figure 5:**
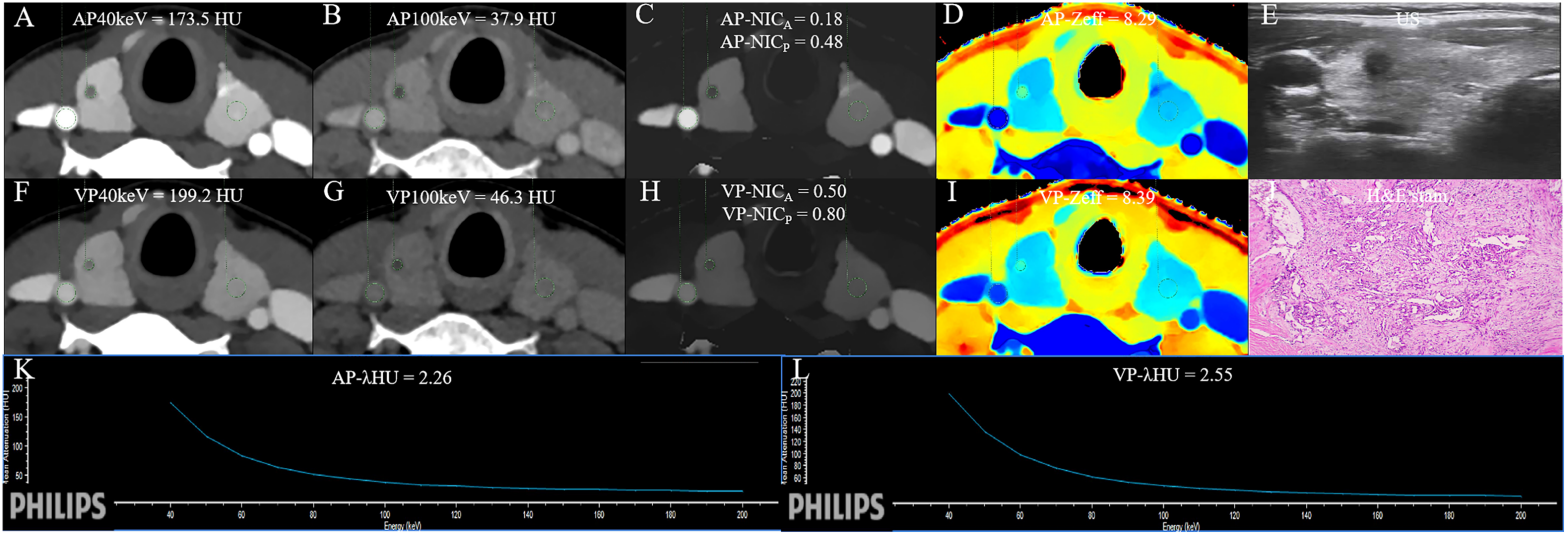
The DLCT quantitative parameters and Haematoxylin-eosin stain in a 37-year-old woman with thyroid papillary carcinoma who tested positive for HT and negative for BRAF. **(A, B)** CT value of arterial phase 40 keV and 100 keV monochromatic images is 173.5 HU, and 37.9 HU, respectively. **(C)** Arterial phase NIC_A_ and NIC_P_ are 0.18, and 0.48, respectively. **(D)** Arterial phase Z_eff_ is 8.29. **(E)** US images. **(F, G)** CT value of venous phase 40 keV and 100 keV monochromatic image is 199.2 HU, and 46.3HU, respectively. **(H)** Venous phase NIC_A_ and NIC_P_ are 0.50, and 0.80, respectively. **(I)** Venous phase Zeff is 8.39. **(J)** Photomicrograph confirmed the pathological finding of the nodule as papillary thyroid carcinoma. (Hematoxylin-eosin stain; original magnification, 40). **(K)** Arterial phase λ_HU_ of the energy curve is 2.26 HU/keV. **(L)** Venous phase λ_HU_ of the energy curve is 2.55 HU/keV. HT, Hashimoto’s thyroiditis; AP, arterial phase; VP, venous phase; HU, CT value; λ_HU_, the slope of spectral HU curve; IC_nodule_, iodine concentration of thyroid nodule; NIC_P,_ IC_nodule/_IC_thyroid parenchyma_; NIC_A,_ IC_nodule_/IC_carotid artery;_ λ_HU_, slope of the spectral Hounsfield unit curve; Z_eff_, effective atomic number; US, Ultrasound; H&E, Haematoxylin-eosin.

**Figure 6:**
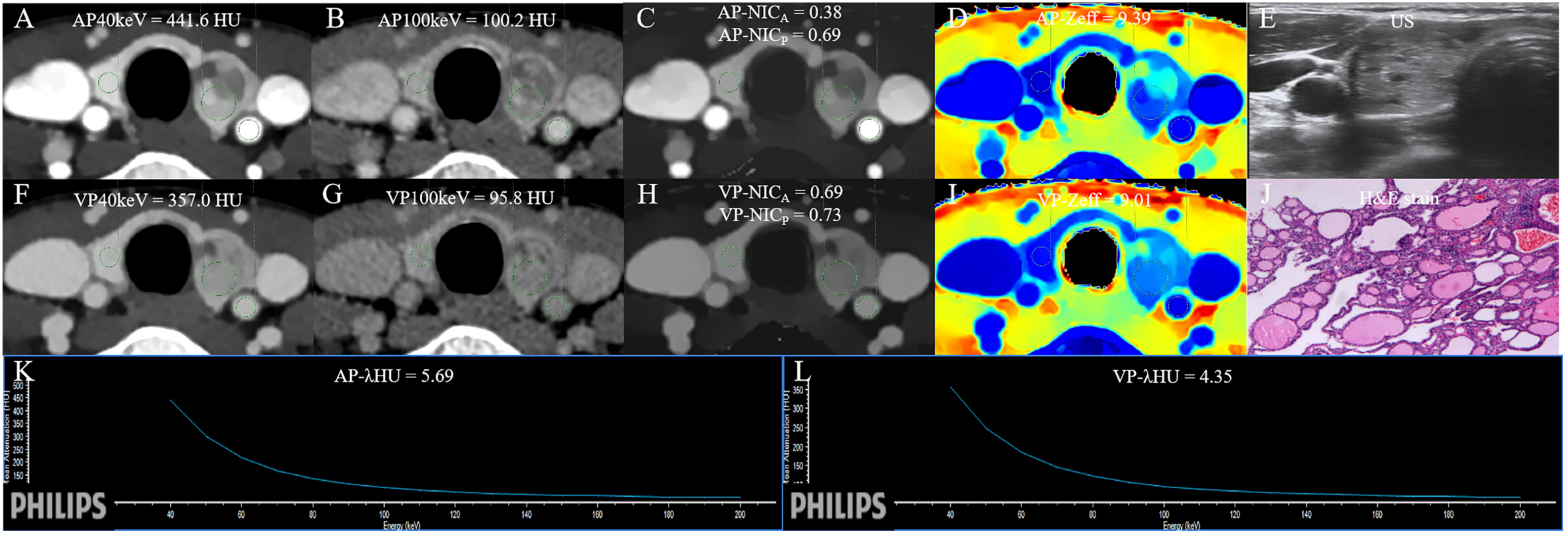
The DLCT quantitative parameters and Haematoxylin-eosin stain in a 47-year-old woman with nodular goiter who tested negative for HT and positive for BRAF. **(A, B)** CT value of arterial phase 40 keV and 100 keV monochromatic image is 441.6 HU, and 100.2 HU, respectively. **(C)** Arterial phase NIC_A_ and NIC_P_ are 0.38, and 0.69, respectively. **(D)** Arterial phase Z_eff_ is 9.39. **(E)** US images. **(F, G)** CT value of venous phase phase 40 keV and 100 keV monochromatic image is 357.0 HU, and 95.8 HU, respectively. **(H)** Venous phase NIC_A_ and NIC_P_ are 0.69, and 0.73, respectively. **(I)** Venous phase Z_eff_ is 9.01. **(J)** Photomicrograph confirmed the pathological finding of the nodule as nodular goiter. (Hematoxylin-eosin stain; original magnification, 40). **(K)** Arterial phase λ_HU_ of the energy curve is 5.69 HU/keV. **(L)** Venous phase λ_HU_ of the energy curve is 4.35 HU/keV. HT, Hashimoto’s thyroiditis; AP, arterial phase; VP, venous phase; HU, CT value; λ_HU_, the slope of spectral HU curve; IC_nodule_, iodine concentration of thyroid nodule; NIC_P,_ IC_nodule/_IC_thyroid parenchyma_; NIC_A,_ IC_nodule_/IC_carotid artery;_ λ_HU_, slope of the spectral Hounsfield unit curve; Z_eff_, effective atomic number; US, Ultrasound; H&E, Haematoxylin-eosin.

The DLCT-clinical nomogram was established according to the DLCT–clinical combined model (**Figure 3**). The formula was as follows: DLCT-clinical nomogram = 2.809 - 5.54 × APNIC_p_ + 1.862 × BRAF V600E + 1.335 × HT. Calibration curve of the nomogram demonstrated good agreement between predicted results and actual observations in both training and validation sets (**Figures 4A, B**). The decision curve analysis revealed that the nomogram provided more clinical benefit than the all-or-none intervention strategy and achieved optimal clinical utility among the three different models in both training and validation sets (**Figures 4C, D**). The DLCT and pathological images from the two examples were shown in **Figures 5** and **6**.

## Discussion

In this study, a DLCT-clinical nomogram comprising AP-NIC_P_, HT, and BRAF V600E demonstrated optimal diagnostic performance for detection of malignant lesions in cytologically indeterminate TN, outperforming both the DLCT model and clinical model in training and validation sets. These findings suggested that the DLCT-clinical nomogram could be a valuable tool for detection of malignant lesions in TNs with indeterminate cytology. Additionally, the relatively objective nature of the DLCT-clinical nomogram made it user-friendly, eliminating the need for experience in identifying typical radiological features, which is especially beneficial for less experienced clinicians.

The study identified that adipose tissue could release cytokines and enhance oxidative stress, thereby promoting the progression of transformed cancer cells (22). However, we found that BMI, extensively used for assessing obesity, with an inability to detect malignant lesions in TNs with indeterminate cytology, which is consistent with previous studies (23–25). In our study, the clinical variables BRAF V600E and HT emerged as important independent predictors of malignant lesions in cytologically indeterminate TN, aligning with findings from prior studies (26–29). BRAF V600E, a member of the Raf family of serine/threonine protein kinases, plays a pivotal role in cell proliferation, differentiation, and apoptosis (30). We propose that cells with BRAF V600E mutation may fail to undergo normal apoptosis, further triggering tumor occurrence (31). HT, an autoimmune thyroid disease, characterized by increased thyroid volume, lymphocyte infiltration of parenchyma, and the presence of antibodies specific to thyroid antigens (32), was associated with an increased probability of thyroid carcinoma in our study. This association may be attributed to lymphocytes stimulating cancer cell proliferation by secreting chemokines and other molecules (33). Moreover, prolonged elevated levels of TSH in HT patients may fuel follicular epithelial proliferation contributing to the development of PTC (32). The clinical model combining BRAF V600E with HT exhibited good sensitivity and relatively poor specificity (0.576 in the training set and 0.545 in the validation set), suggesting that the clinical model had limitations in reducing inappropriate therapy for patients with cytologically indeterminate TNs.

The study further explored the diagnostic values of DLCT parameters and revealed that IC, λ_HU_, NIC_A_, NIC_P,_ and Z_eff_ in the AP and VP were significantly lower in the malignant cohort than in the benign cohort. Multivariable analysis identified AP-NIC_P_ as another independent predictor for malignant TN with indeterminate cytology. Tumor cells can disrupt thyroid follicular cells, which have an iodine uptake function, leading to a reduction in IC of the malignant lesion (34, 35). NICp, calculated based on IC, can provide quantitative information about iodine uptake and tissue perfusion level, superior to IC in overcoming the bias from patient hormone levels (16). Our results indicated that the DLCT model (AP-NICp) was useful for detection of TNs with indeterminate cytology, achieving AUCs of 0.824 and 0.779 in the training and validation sets, respectively. However, accurately identifying TN status remained challenging, with poor specificity (0.667 in the training set and 0.636 in the validation set).

Based on these above results, we established a DLCT-clinical model incorporating the clinical variables (HT and BRAF V600E) with AP-NICp to detect malignant lesions in cytologically indeterminate TN. The DLCT-clinical model demonstrated the best diagnostic utility with an AUC of 0.875 in the training set and an AUC of 0.874 in the validation set. Meanwhile, the combined model exhibited good sensitivity (0.857 in the validation set) and specificity (0.727 in the validation set). Moreover, the DLCT-clinical model was visually presented by a nomogram, serving as a simple tool for clinician use. Typical radiological features were excluded from our study due to the subjectivity of operator dependence. Our proposed nomogram exhibited good agreement with pathological results, as shown in the calibration curves, and demonstrated good clinical utility for patients with cytologically indeterminate TN, as seen in decision curve. We compared the diagnostic performance of DLCT with different examinations (**Supplementary Material**). It showed that the DLCT-clinical nomogram was not inferior to some different examinations. Therefore, the DLCT-clinical nomogram may serve as a simple, objective, and reliable tool to guide clinical personalized assessment.

Studies reported that artificial intelligence algorithms allow for precise quantification of features such as nuclear area and elongation factor or crowding of nuclei, which appear to be differently distributed between benign and malignant nodules, revealing the potential to efficiently evaluate FNA cytology cases (36). The whole slide imaging, a digital pathology modality, facilitates the use of artificial intelligence in the field of pathology (37). In the future study, we will develop an artificial intelligence model with multidimensional features including radiological images, clinical indicators, genetic features, and pathological features to reduce the indeterminate diagnosis of patients with TNs, especially for organ donors who might contribute to the transmission of thyroid cancer (38).

Indeed, this study has some limitations. Firstly, it is a single-center retrospective study with a relatively small sample size. Despite perfect internal validation, the built DLCT-clinical nomogram is not yet suitable for general use before external validation of the predictive nomogram. Thus, multicenter and external prospective studies with larger sample sizes are needed to further validate the diagnostic power of the nomogram. Secondly, since the malignant TNs in our study were papillary thyroid carcinomas, the efficacy of our nomogram may be limited in identifying other pathological types of malignant TNs.

## Conclusion

In conclusion, this study provides a DLCT-clinical nomogram, consisting of AP-NIC_P_, HT, and BRAF V600E, which demonstrates favorable performance in detecting malignant lesions in cytologically indeterminate TNs. This validated nomogram can be an effective tool to assist clinicians in assessing the nature of cytologically indeterminate TN and reduce unbeneficial management.

## Data availability statement

The raw data supporting the conclusions of this article will be made available by the authors, without undue reservation.

## Ethics statement

The studies involving humans were approved by the Review Committee of Chongqing General Hospital. The studies were conducted in accordance with the local legislation and institutional requirements. Written informed consent for participation was not required from the participants or the participants’ legal guardians/next of kin in accordance with the national legislation and institutional requirements.

## Author contributions

XR: Writing – original draft, Writing – review & editing, Methodology. JZ: Data curation, Writing – original draft, Writing – review & editing. ZS: Methodology, Supervision, Visualization, Writing – original draft, Writing – review & editing. QL: Methodology, Writing – original draft, Writing – review & editing. DZh: Data curation, Funding acquisition, Writing – original draft, Writing – review & editing. XL: Data curation, Writing – original draft, Writing – review & editing. JY: Data curation, Methodology, Writing – original draft, Writing – review & editing. ZL: Data curation, Writing – original draft, Writing – review & editing. YW: Data curation, Writing – original draft, Writing – review & editing. DZe: Data curation, Writing – original draft, Writing – review & editing. XZ: Writing – original draft, Writing – review & editing. ZT: Conceptualization, Funding acquisition, Supervision, Writing – original draft, Writing – review & editing.

## References

[1] Durante C Grani G Lamartina L Filetti S Mandel SJ Cooper DS. The diagnosis and management of thyroid nodules: A review. JAMA (2018) 319(9):914–24. doi: 10.1001/jama.2018.0898 29509871

[2] Haugen BR Alexander EK Bible KC Doherty GM Mandel SJ Nikiforov YE. 2015 American thyroid association management guidelines for adult patients with thyroid nodules and differentiated thyroid cancer: the american thyroid association guidelines task force on thyroid nodules and differentiated thyroid cancer. Thyroid. (2016) 26:1–133. doi: 10.1089/thy.2015.0020 26462967 PMC4739132

[3] Nikiforov YE Carty SE Chiosea SI Coyne C Duvvuri U Ferris RL. Impact of the multi-gene thyroSeq next-generation sequencing assay on cancer diagnosis in thyroid nodules with atypia of undetermined significance/follicular lesion of undetermined significance cytology. Thyroid. (2015) 25:1217–23. doi: 10.1089/thy.2015.0305 PMC4652198 26356635

[4] Cibas ES Ali SZ. The 2017 bethesda system for reporting thyroid cytopathology. Thyroid. (2017) 27:1341–6. doi: 10.1089/thy.2017.0500 29091573

[5] Vriens D de Wilt JH van der Wilt GJ Netea-Maier RT Oyen WJ de Geus-Oei LF. The role of [18F]-2-fluoro-2-deoxy-d-glucose-positron emission tomography in thyroid nodules with indeterminate fine-needle aspiration biopsy: systematic review and meta-analysis of the literature. Cancer. (2011) 117:4582–94. doi: 10.1002/cncr.26085 21432844

[6] Merten MM Castro MR Zhang J Durski J Ryder M. Examining the role of preoperative positron emission tomography/computerized tomography in combination with ultrasonography in discriminating benign from Malignant cytologically indeterminate thyroid nodules. Thyroid. (2017) 27:95–102. doi: 10.1089/thy.2016.0379 27762671

[7] Bakkar S Macerola E Proietti A Aljarrah Q Al-Omar K Materazzi G. Developing a tool that could reliably refute total thyroidectomy for solitary Bethesda IV thyroid nodules. Updates Surg. (2021) 73:281–8. doi: 10.1007/s13304-020-00783-w 32410160

[8] Piccardo A Puntoni M Treglia G Foppiani L Bertagna F Paparo F. Thyroid nodules with indeterminate cytology: prospective comparison between 18F-FDG-PET/CT, multiparametric neck ultrasonography, 99mTc-MIBI scintigraphy and histology. Eur J Endocrinol. (2016) 174:693–703. doi: 10.1530/EJE-15-1199 26966173

[9] Patel KN Yip L Lubitz CC Grubbs EG Miller BS Shen W. The american association of endocrine surgeons guidelines for the definitive surgical management of thyroid disease in adults. Ann Surg. (2020) 271:e21–93. doi: 10.1097/SLA.0000000000003580 32079830

[10] Nemec U Nemec SF Novotny C Weber M Czerny C Krestan CR. Quantitative evaluation of contrast-enhanced ultrasound after intravenous administration of a microbubble contrast agent for differentiation of benign and Malignant thyroid nodules: assessment of diagnostic accuracy. Eur Radiol. (2012) 22:1357–65. doi: 10.1007/s00330-012-2385-6 22322310

[11] Liu X Ouyang D Li H Zhang R Lv Y Yang A. Papillary thyroid cancer: dual-energy spectral CT quantitative parameters for preoperative diagnosis of metastasis to the cervical lymph nodes. Radiology. (2015) 275:167–76. doi: 10.1148/radiol.14140481 25521777

[12] Sparano C Verdiani V Pupilli C Perigli G Badii B Vezzosi V. Choosing the best algorithm among five thyroid nodule ultrasound scores: from performance to cytology sparing-a single-center retrospective study in a large cohort. Eur Radiol. (2021) 31:5689–98. doi: 10.1007/s00330-021-07703-5 PMC8270877 33599836

[13] Piccardo A Puntoni M Dezzana M Bottoni G Foppiani L Marugo A. Indeterminate thyroid nodules. The role of 18F-FDG PET/CT in the “era” of ultrasonography risk stratification systems and new thyroid cytology classifications. Endocrine. (2020) 69:553–61. doi: 10.1007/s12020-020-02239-y 32124261

[14] Johnson TR Krauss B Sedlmair M Grasruck M Bruder H Morhard D. Material differentiation by dual energy CT: initial experience. Eur Radiol. (2007) 17:1510–7. doi: 10.1007/s00330-006-0517-6 17151859

[15] Hamid S Nasir MU So A Andrews G Nicolaou S Qamar SR. Clinical applications of dual-energy CT. Korean J Radiol. (2021) 22:970–82. doi: 10.3348/kjr.2020.0996 PMC8154785 33856133

[16] Forghani R. An update on advanced dual-energy CT for head and neck cancer imaging. Expert Rev Anticancer Ther. (2019) 19:633–44. doi: 10.1080/14737140.2019.1626234 31177872

[17] Al-Najami I Mahmoud Sheta H Baatrup G. Differentiation between Malignant and benign rectal tumors by dual-energy computed tomography - a feasibility study. Acta Oncol. (2019) 58:S55–9. doi: 10.1080/0284186X.2019.1574404 30764692

[18] Wang P Tang Z Xiao Z Hong R Wang R Wang Y. Dual-energy CT in differentiating benign sinonasal lesions from Malignant ones: comparison with simulated single-energy CT, conventional MRI, and DWI. Eur Radiol. (2022) 32:1095–105. doi: 10.1007/s00330-021-08159-3 34427744

[19] Song Z Li Q Zhang D Li X Yu J Liu Q. Nomogram based on spectral CT quantitative parameters and typical radiological features for distinguishing benign from Malignant thyroid micro-nodules. Cancer Imaging. (2023) 23:13. doi: 10.1186/s40644-023-00525-2 36703218 PMC9878766

[20] Wu YY Wei C Wang CB Li NY Zhang P Dong JN. Preoperative prediction of cervical nodal metastasis in papillary thyroid carcinoma: value of quantitative dual-energy CT parameters and qualitative morphologic features. AJR Am J Roentgenol. (2021) 216:1335–43. doi: 10.2214/AJR.20.23516 33760651

[21] Zhang T Hou F Liu D Zhou H Sun Y Deng X. Association of Hashimoto’s thyroiditis and anti-thyroid antibodies with oral lichen planus: A cross-sectional study. Front Immunol. (2022) 13:967988. doi: 10.3389/fimmu.2022.967988 36052085 PMC9424685

[22] Jovanović M Kovačević S Brkljačić J Djordjevic A. Oxidative stress linking obesity and cancer: is obesity a ‘Radical trigger’ to cancer? Int J Mol Sci. (2023) 24:8452. doi: 10.3390/ijms24098452 37176160 PMC10179114

[23] Ahmadi S Pappa T Kang AS Coleman AK Landa I Marqusee E. Point of care measurement of body mass index and thyroid nodule Malignancy risk assessment. Front Endocrinol (Lausanne). (2022) 13:824226. doi: 10.3389/fendo.2022.824226 35222281 PMC8873520

[24] Fussey JM Beaumont RN Wood AR Vaidya B Smith J Tyrrell J. Does obesity cause thyroid cancer? A mendelian randomization study. J Clin Endocrinol Metab. (2020) 105:e2398–407. doi: 10.1210/clinem/dgaa250 PMC7274488 32392279

[25] Stansifer KJ Guynan JF Wachal BM Smith RB. Modifiable risk factors and thyroid cancer. Otolaryngol Head Neck Surg. (2015) 152:432–7. doi: 10.1177/0194599814564537 25552593

[26] Chen X Zhou Q Wang F Zhang F Du H Zhang Q. Value of BRAF V600E in high-risk thyroid nodules with benign cytology results. AJNR Am J Neuroradiol. (2018) 39:2360–5. doi: 10.3174/ajnr.A5898 PMC7655409 30498021

[27] Zarkesh M Zadeh-Vakili A Akbarzadeh M Nozhat Z Fanaei SA Hedayati M. BRAF V600E mutation and microRNAs are helpful in distinguishing papillary thyroid Malignant lesions: Tissues and fine needle aspiration cytology cases. Life Sci. (2019) 223:166–73. doi: 10.1016/j.lfs.2019.03.034 30890403

[28] Mao L Zheng C Ou S He Y Liao C Deng G. Influence of Hashimoto thyroiditis on diagnosis and treatment of thyroid nodules. Front Endocrinol (Lausanne). (2022) 13:1067390. doi: 10.3389/fendo.2022.1067390 36619577 PMC9816323

[29] Nicolson NG Brown TC Korah R Carling T. Immune cell infiltrate-associated dysregulation of DNA repair machinery may predispose to papillary thyroid carcinogenesis. Surgery. (2020) 167:66–72. doi: 10.1016/j.surg.2019.02.024 31439400

[30] de Koster EJ de Geus-Oei LF Dekkers OM van Engen-van Grunsven I Hamming J Corssmit EPM. Diagnostic utility of molecular and imaging biomarkers in cytological indeterminate thyroid nodules. Endocr Rev. (2018) 39:154–91. doi: 10.1210/er.2017-00133 29300866

[31] Huang G Chen J Zhou J Xiao S Zeng W Xia J. Epigenetic modification and BRAF gene mutation in thyroid carcinoma. Cancer Cell Int. (2021) 21:687. doi: 10.1186/s12935-021-02405-w 34923978 PMC8684614

[32] Ralli M Angeletti D Fiore M D’Aguanno V Lambiase A Artico M. Hashimoto’s thyroiditis: An update on pathogenic mechanisms, diagnostic protocols, therapeutic strategies, and potential Malignant transformation. Autoimmun Rev. (2020) 19:102649. doi: 10.1016/j.autrev.2020.102649 32805423

[33] Vita R Ieni A Tuccari G Benvenga S. The increasing prevalence of chronic lymphocytic thyroiditis in papillary microcarcinoma. Rev Endocr Metab Disord. (2018) 19:301–9. doi: 10.1007/s11154-018-9474-z 30456477

[34] Kogai T Hershman JM Motomura K Endo T Onaya T Brent GA. Differential regulation of the human sodium/iodide symporter gene promoter in papillary thyroid carcinoma cell lines and normal thyroid cells. Endocrinology. (2001) 142:3369–79. doi: 10.1210/endo.142.8.8344 11459780

[35] Liou MJ Lin JD Chan EC Liu FH Chao TC Weng HF. Detection of mRNA of sodium iodide symporter in benign and Malignant human thyroid tissues. Cancer Lett. (2000) 160:75–80. doi: 10.1016/s0304-3835(00)00565-6 11098087

[36] Girolami I Marletta S Pantanowitz L Torresani E Ghimenton C Barbareschi M. Impact of image analysis and artificial intelligence in thyroid pathology, with particular reference to cytological aspects. Cytopathology. (2020) 31:432–44. doi: 10.1111/cyt.12828 32248583

[37] Marletta S Salatiello M Pantanowitz L Bellevicine C Bongiovanni M Bonoldi E. Delphi expert consensus for whole slide imaging in thyroid cytopathology. Cytopathology. (2023) 34:581–9. doi: 10.1111/cyt.13279 37530465

[38] Huang H Zhu MJ Gao Q Huang YL Li WM. Comparison of diagnostic values of ACR TI-RADS versus C-TIRADS scoring and classification systems for the elderly thyroid cancers. Int J Gen Med. (2023) 16:4441–51. doi: 10.2147/IJGM.S429681 PMC10546997 37795310

