# Supplementary material for: Detection of malignant lesions in cytologically indeterminate thyroid nodules using a dual-layer spectral detector CT-clinical nomogram
Source: Front Oncol. 2024 May 28;14:1357419. doi: 10.3389/fonc.2024.1357419 (PMC11165073; doi:10.3389/fonc.2024.1357419)
Supplement: Supplementary file 1 [file Table_1.docx]

**Supplementary Material**

Table 1: Performance of different tools for detecting malignancy in cytologically indeterminate thyroid nodules

| **Author ( year)** | **Country** | **Study design** | **Reference standards** | **Sample size**  **(benignity: malignity)** | **Demographic information** | **examination** | **AUC** | **Sensitivity** | **specificity** |
| --- | --- | --- | --- | --- | --- | --- | --- | --- | --- |
| Ciappuccini R^(1)^  (2021) | France | retrospective, 1‐institution | Pathology | 107  (87:20) | 75% women, 55±14 years | 18F-Fluorocholine PET/CT | N/A | 0.900 | 0.500 |
| Sengul D^(2)^  (2019) | Turkey | retrospective, 1‐institution | Pathology | 122 (110:12） | 77.9% women, 1.88±12.01years | strain elastography | 0.892 | 0.980 | 0.700 |
| Barbosa TLM^(3)^  (2019) | Brazil | retrospective, 1‐institution | Pathology | 140  (74:66） | 84.3% women,  49±13years | ATA US pattern | N/A | 0.953 | 0.846 |
| He YP^(4)^  (2017) | China | retrospective, 1‐institution | Pathology | 453 (198:255) | 80.1% women, N/A | TI-RADS, US features | 0.731 | 0.976 | 0.485 |
| Zhou L^(5)^  (2023) | China | retrospective, 1‐institution | Pathology | 159  (38:121) | 72.3% women, 46.09±12.09 years | S-Detect (an image-analytic program) | 0.795 | 0.959 | 0.881 |
| Guan X^(6)^  (2023) | China | retrospective, 1‐institution | Pathology | 87  （18:69） | 79.1% women,  48±12.7years | Blood tests, molecular tests, repeat FNAB | 0.837 | 0.841 | 0.833 |
| D'Andréa G^(7)^  (2023) | France | retrospective,  4‐institutions | Pathology | 1335  （862:437) | 78% women, 52.9±14.5 years | clinical, biological, US, and cytological data | 0.840 | 0.573 | 0.900 |

AUC, area under receiver operating characteristic curve; N/A, Not applicable; PET/CT, positron emission tomography/ computed tomography; ATA, American Thyroid Association guidelines; US, Ultrasound; TI-RADS, Thyroid Imaging Reporting and Data System; FNAB, Fine-needle aspiration biopsy.

Reference

1. Ciappuccini R, Licaj I, Lasne-Cardon A, Babin E, de Raucourt D, Blanchard D, et al. F-Fluorocholine Positron Emission Tomography/Computed Tomography is a Highly Sensitive but Poorly Specific Tool for Identifying Malignancy in Thyroid Nodules with Indeterminate Cytology: The Chocolate Study. Thyroid (2021)31:800-809. doi: 10.1089/thy.2020.0555
2. Sengul D, Sengul I, Van Slycke S. Risk stratification of the thyroid nodule with Bethesda indeterminate cytology, category III, IV, V on the one surgeon-performed US-guided fine-needle aspiration with 27-gauge needle, verified by histopathology of thyroidectomy: the additional value of one surgeon-performed elastography. Acta Chir Belg (2019)119(1):38-46. doi: 10.1080/00015458.2018.1551769
3. Junior COM, Graf H, Cavalvanti T, Trippia MA, da Silveira Ugino RT, de Oliveira GL, et al. ACR TI-RADS and ATA US scores are helpful for the management of thyroid nodules with indeterminate cytology. BMC Endocr Disord (2019)19(1):112. doi: 10.1186/s12902-019-0429-5
4. He YP, Xu HX, Zhao CK, Sun LP, Li XL, Yue WW, et al. Cytologically indeterminate thyroid nodules: increased diagnostic performance with combination of US TI-RADS and a new scoring system. Sci Rep (2017)31;7:6906. doi: 10.1038/s41598-017-07353-y
5. Zhou L, Zheng LL, Zhang CJ, Wei HF, Xu LL, Zhang MR, et al. Comparison of S-Detect and thyroid imaging reporting and data system classifications in the diagnosis of cytologically indeterminate thyroid nodules. Front Endocrinol (Lausanne)(2023)24;14:1098031. doi: 10.3389/fendo.2023.1098031
6. Guan X, Yu T, Zhang Z, Chen L, Yan A, Li Y, et al. Risk assessment of cytologically indeterminate thyroid nodules with integrated molecular testing and repeat biopsy: a surgical decision-oriented tool. World J Surg Oncol (2023);21:34. doi: 10.1186/s12957-023-02917-x
7. D'Andréa G, Gal J, Mandine L, Dassonville O, Vandersteen C, Guevara N, Castillo L, Poissonnet G, Culié D, Elaldi R, Sarini J, Decotte A, Renaud C, Vergez S, Schiappa R, Chamorey E, Château Y, Bozec A.et al Application of machine learning methods to guide patient management by predicting the risk of malignancy of Bethesda III-V thyroid nodules. Eur J Endocrinol(2023)188(3):lvad017. doi: 10.1093/ejendo/lvad017
